# Establishment and function of the rhizosphere fungal community in rare and endangered plant *Alsophila spinulosa*

**DOI:** 10.3389/fmicb.2025.1552748

**Published:** 2025-05-21

**Authors:** Zhang Hui-Min, Yan Ling-Bin, Yuan Dong-Mei, Liu Feng, Cao Xiu-Gang, Wang Dai-Yan, He Qin-Qin, Mu Jun, Yu Li-Fei, Liu Yuan, Chen Zhi-Fei

**Affiliations:** ^1^The Key Laboratory of Plant Resources Conservation and Germplasm Innovation in Mountainous Region (Ministry of Education), College of Life Sciences, Guizhou University, Guiyang, China; ^2^Research Center of Karst Ecological Environment of Guizhou University, Guiyang, China; ^3^Chishui Alsophila National Nature Reserve Administration, Chishui, China; ^4^Guizhou Xishui National Nature Reserve Administration, Xishui, China

**Keywords:** *Alsophila spinulosa*, fungal diversity, rhizosphere fungal, FUNGuild, community assembly

## Abstract

**Introduction:**

The “rhizosphere effect” in plants occurs within approximately 5 mm from the root surface, where microbial communities exhibit distinct species composition and structural characteristics compared to non-rhizospheric soil. Root-associated fungi play crucial roles in nutrient acquisition enhancement, stress resistance improvement, organic matter decomposition, and carbon cycle promotion. Current research shows limited understanding of the rhizospheric fungal communities in *Alsophila spinulosa*, a rare and endangered plant species.

**Methods:**

This study conducted a comparative analysis of fungal community composition and structural differences between *A. spinulosa* rhizospheric and non-rhizospheric soils. The functional roles of fungi in these distinct communities were systematically analyzed, with particular emphasis on identifying keystone fungal taxa within the rhizosphere, and explained the construction process of fungal communities.

**Results:**

The results showed that there was no difference in α diversity between the rhizosphere and non-rhizosphere soil fungal communities of *A. spinulosa*, but the β diversity was significantly differentiated, indicating that the difference between rhizosphere and non-rhizosphere fungal communities was mainly reflected in species composition rather than species number. The two communities have common dominant phylum: Ascomycetes and Basidiomycetes, and common dominant genera: *Mortierella* and *Saitozyma*. The functional type was mainly saprotic. Linear discriminant analysis effect size (LEfSe) analysis revealed four biomarker genera (*Arthopyrenia*, *Hypochnicium*, *Tremella*, and *Syncephalis*) enriched in the *A. spinulosa* rhizospheric fungal community. Venn diagram analysis identified 169 core genera within this community, with *Flavodon* exclusively present in the rhizosphere. Mechanistic analysis of community assembly demonstrated that stochastic processes predominantly governed the structuring of rhizospheric fungal communities.

**Discussion:**

In conclusion, this study elucidates the functional composition and assembly mechanisms of rhizospheric fungal communities in *A. spinulosa*, while identifying keystone fungal taxa potentially critical to its survival. Future investigations should: Quantify the specific contribution of *Flavodon* to *A. spinulosa*; Decipher the mechanistic linkages between these fungi and the plant’s stress resistance traits; Implement plant–soil-microbe synergistic restoration strategies to enhance natural regeneration capacity of *A. spinulosa* populations.

## 1 Introduction

*Alsophila spinulosa* is one of the few remaining rare woody fern species and represents an ancient relict plant that coexisted with dinosaurs (Zong et al., 2016). This species holds significant value not only for its medicinal properties but also for studies on speciation, phytogeography, paleobiology, and paleoenvironmental changes (Chen et al., 2008). However, *A. spinulosa* populations face severe threats due to short spore viability, difficult germination, prolonged life cycles, strict habitat requirements, and anthropogenic logging, necessitating urgent conservation measures (Cheng et al., 1990).

The rhizosphere is a micro-domain environment affected by plant root activity, ranging from 2 to 3 mm from the surface of fine roots. The interactions among plants, soil, and microorganisms in this area is very complicated (Zhou, 2011; Zhou et al., 2014). The roots of seed plants can act on the rhizosphere microbial flora by secreting sugars, amino acids, organic acids, and other substances, thus affecting the structure of the rhizosphere microbial community (Yuan et al., 2018; Huang et al., 2022). The composition and diversity of rhizosphere microbial species can also affect plant growth and development (Zhalnina et al., 2018). Of these species, rhizosphere soil fungi play an important role in regulating nutrient intake, promoting growth and development, improving the disease resistance of host plants, and maintaining the balance of rhizosphere microecosystems (Hannula et al., 2020; Powell and Rillig, 2018).

Compared with seed plants, the fibrous root structure of ferns is relatively simple, which is usually an adventitious root on the stem or bud, with no true apical meristem, underdeveloped vascular tissue, and low absorption and transport capacity (Dong et al., 2015; Sherwin Carlquist, 2001; Zhang, 2012). *A. spinulosa*, the only surviving woody fern today, has lignified main stems that can reach 6 m or more in height, but its absorption root anatomical characteristics are not significantly different from those of some small herbaceous ferns (Xiang et al., 2022). Research on ferns mainly focuses on vesicular-arbuscular (VA) mycorrhiza in the rhizosphere soil of ferns (Sha et al., 1998; Zhao, 1998). It has been suggested that VA mycorrhiza play a role in the nutrition and adaptation of ferns. This has been demonstrated in the fern *Osmunda japonica* (Ma and He, 2010), but not in *A. spinulosa.* Studies on the rhizosphere microorganisms of *A. spinulosa* have shown that bacterial community composition and diversity were greatly influenced by soil pH, while fungal community composition and diversity were greatly influenced by available phosphorus, organic carbon, sucrase, and urease (Che et al., 2024). Endophytic fungi in *A. spinulosa* exhibited higher diversity in leaf rachises compared to leaf blades (Zang et al., 2020). Nevertheless, the functional roles and assembly mechanisms of rhizospheric fungal communities in *A. spinulosa* remain insufficiently investigated, representing a critical research gap in understanding this relict species’ microbiome ecology. This study contributes to elucidating the unique rhizosphere microbiome assembly mechanisms in the endangered *A. spinulosa*, addressing a critical knowledge gap in microbial ecology of non-model plants. Simultaneously, it provides a microbiological foundation for developing targeted conservation strategies for this species.

## 2 Materials and methods

### 2.1 Study site

The Guizhou Chishui *A. spinulosa* National Nature Reserve is located in Chishui, Guizhou, China, with a latitudinal range of 28°20′19″–28°28′40″ N and a longitudinal range of 105°57′54″–106°7′7″ E″. It has obvious Danxia landform characteristics and a large number of *A. spinulosa* forest areas around the world (Liu, 2018). The reserve belongs to the middle subtropical humid monsoon climate zone, and the river valley has characteristics similar to the southern subtropical climate. There is no severe cold in winter, no scorching heat in summer, less sunshine, high temperature, high humidity, abundant precipitation, many cloudy and rainy days, and large vertical differences (Yang et al., 2011).

### 2.2 Sample collection

In the reserve, Jinfeng Mountain (JFS), Mentouxi Gou (MTXG), and Xujia Gou (XJG), which are densely distributed and less populated by human beings, were selected as sample plots. Three *A. spinulosa* individuals with good growth conditions and similar growth were randomly selected from different orientations in each plot, within a 0.5-meter radius centered on the main stem, surface soil was removed to expose *A. spinulosa* roots. Following the detachment of larger soil aggregates, the remaining soil adhering to the root system was collected as rhizosphere soil (R). Non-rhizosphere soil samples were collected from areas beyond the 0.5-meter radius (at depths of 0–20 cm) and at least 10 cm away from any visible roots (NR) (Riley and Barber, 1970). After removing impurities, such as stones, plant residues, and roots, the collected samples were quickly placed into sterile centrifuge tubes, numbered, and stored in liquid nitrogen. The samples collected in the same plot were divided into 2 groups according to rhizosphere or non-rhizosphere soil, and 18 soil samples from 6 groups were collected in 3 plots.

### 2.3 DNA extraction and sequencing

After genomic DNA was extracted from the sample, the quality of the extracted genomic DNA was detected using 1% agarose gel electrophoresis, and the DNA concentration and purity were determined using NanoDrop2000 (Thermo Scientific Company, Waltham, Massachusetts, United States). The ITS2 region was amplified using the primers ITS1F (5′-CTTGGTCATTTAGAGGAAGTAA-3′) and ITS2R (5′-GCTGCGTTCTTCATCGATGC-3′) using the extracted DNA as a template. The PCR products of the same sample were mixed and tested using 2% agarose gel electrophoresis. The PCR products were cut and recovered using an AchyPrep DNA Gel Recovery Kit (AXYGEN Company, San Carlos, California, United States), eluted by Tris_HCl, and tested by 2% agarose electrophoresis. Based on the preliminary quantitative results of electrophoresis, the PCR products were detected and quantified using the QuantiFluor^™^-ST Blue Fluorescence Quantification System (Promega Company, Madison, Wisconsin, United States). Purified amplicons were pooled in equimolar amounts and paired-end sequenced on the Illumina Nextseq2000 Platform (Illumina, San Diego, California, United States) according to the standard protocols of Majorbio Bio-Pharm Technology Co., Ltd. (Shanghai, China). The raw sequencing reads were deposited into the NCBI Sequence Read Archive (SRA) database (Accession Number: PRJNA1033401).

### 2.4 Data analysis

Differences between sample groups were tested using the Kruskal–Wallis test. Principal coordinates analysis (PCoA) based on the Bray–Curtis distance was used to reveal the differences and similarities between the two groups of samples. Functional classification of fungi was carried out using the FUNGuild database. The linear discriminant analysis (LDA) effect size (LEfSe) was performed to identify the significantly abundant taxa (phylum to genera) of fungi among the different groups (LDA score > 3, *p* < 0.05). Venn diagrams were used to show the number and proportion of common and endemic fungi among groups. A phylogenetic tree was used to reveal the genetic relationships between each species in the sample during evolution. Employed a null model approach with 999 randomizations to calculate the beta nearest taxon index (βNTI), thereby evaluating the relative contributions of deterministic and stochastic processes in microbiome assembly. All statistical analyses were done with the free R software, version 2023.9.1.494.

## 3 Results

### 3.1 Fungal abundance and community functional composition

The sequencing data were spliced and underwent quality control and de-ligation to obtain an optimized sequence with an average length of 232 bp. The operational taxonomic unit (OTU) abundance table was obtained by clustering the optimized sequences at a similarity level of 97%. Sequences aligned to chloroplasts and mitochondria were removed, and the ITS database was used to annotate species information. At the phylum and genus levels, the abundance of fungal communities in the rhizosphere soil was lower than that in the non-rhizosphere soil, but there were no significant differences (Table 1).

**Table 1:** Number of fungi in different taxa.

Taxon	Rhizosphere	Non-rhizosphere	Total
Sequences	438,085	462,269	900,354
OTUs	5,322	5,315	7,636
Species	867	893	1,091
Genus	561	579	677
Family	270	268	200
Order	114	118	129
Class	49	50	56
Phylum	12	14	14

OTUs refer to the operational taxonomic units.

**Figure 1:**
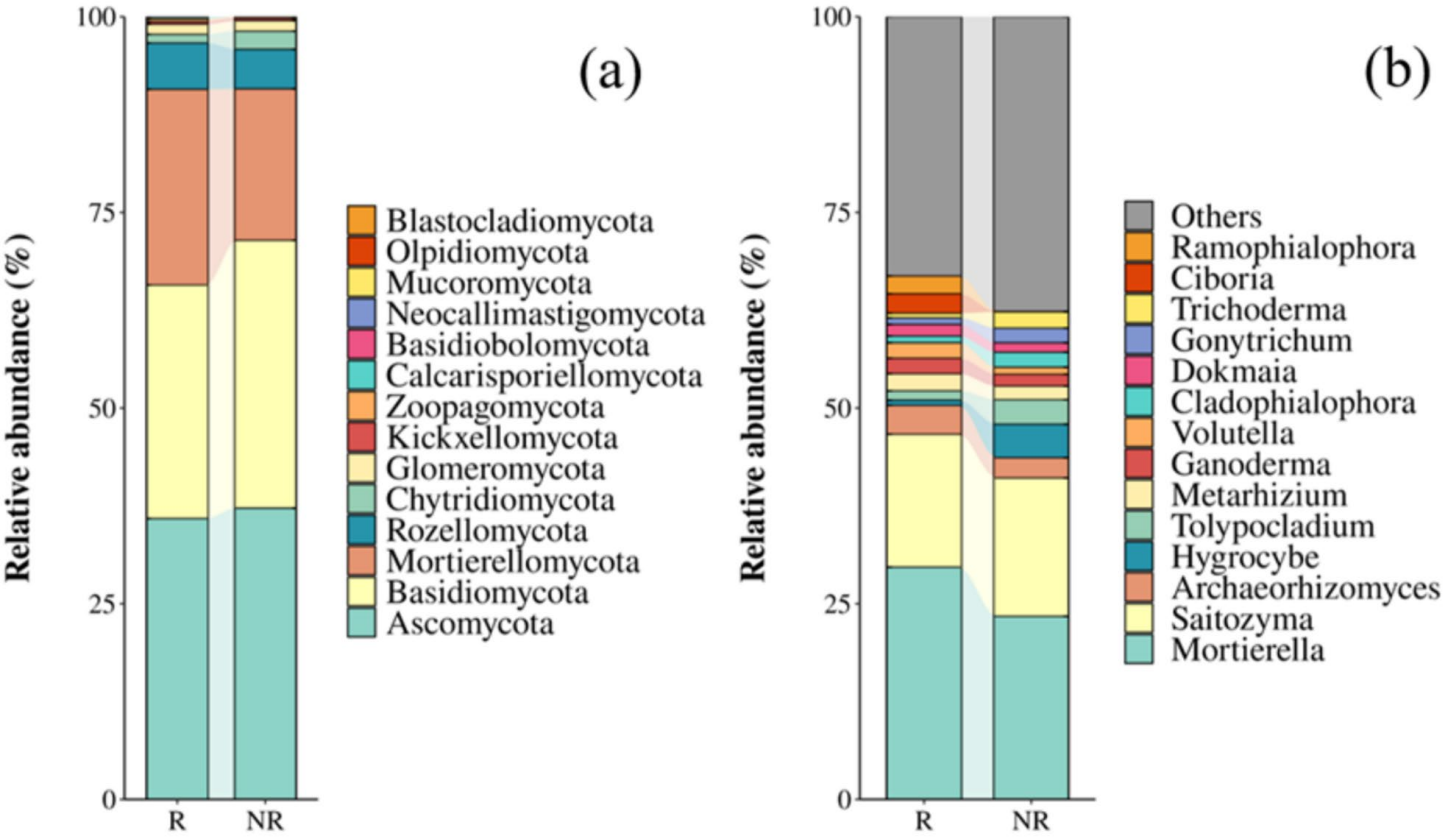
Relative abundance of fungal communities at the phylum **(a)** and genus **(b)** levels. R: rhizosphere fungal community; NR: non-rhizosphere fungal community.

Ascomycota and Basidiomycota were the most dominant phyla in both the rhizosphere and non-rhizosphere fungal communities (Figure 1a), and *Mortierella* and *Saitozyma* were dominant genera common between the two communities (Figure 1b).

Based on different nutritional methods, fungi were divided into 3 categories: pathotrophs, symbiotrophs, and saprotrophs. Based on these categories, fungi were divided into several guilds. Of the phyla and genera, 71.43% and 76.35%, respectively, were successfully annotated, and the credibility was assessed as “probable” and “highly probable.”

**Figure 2:**
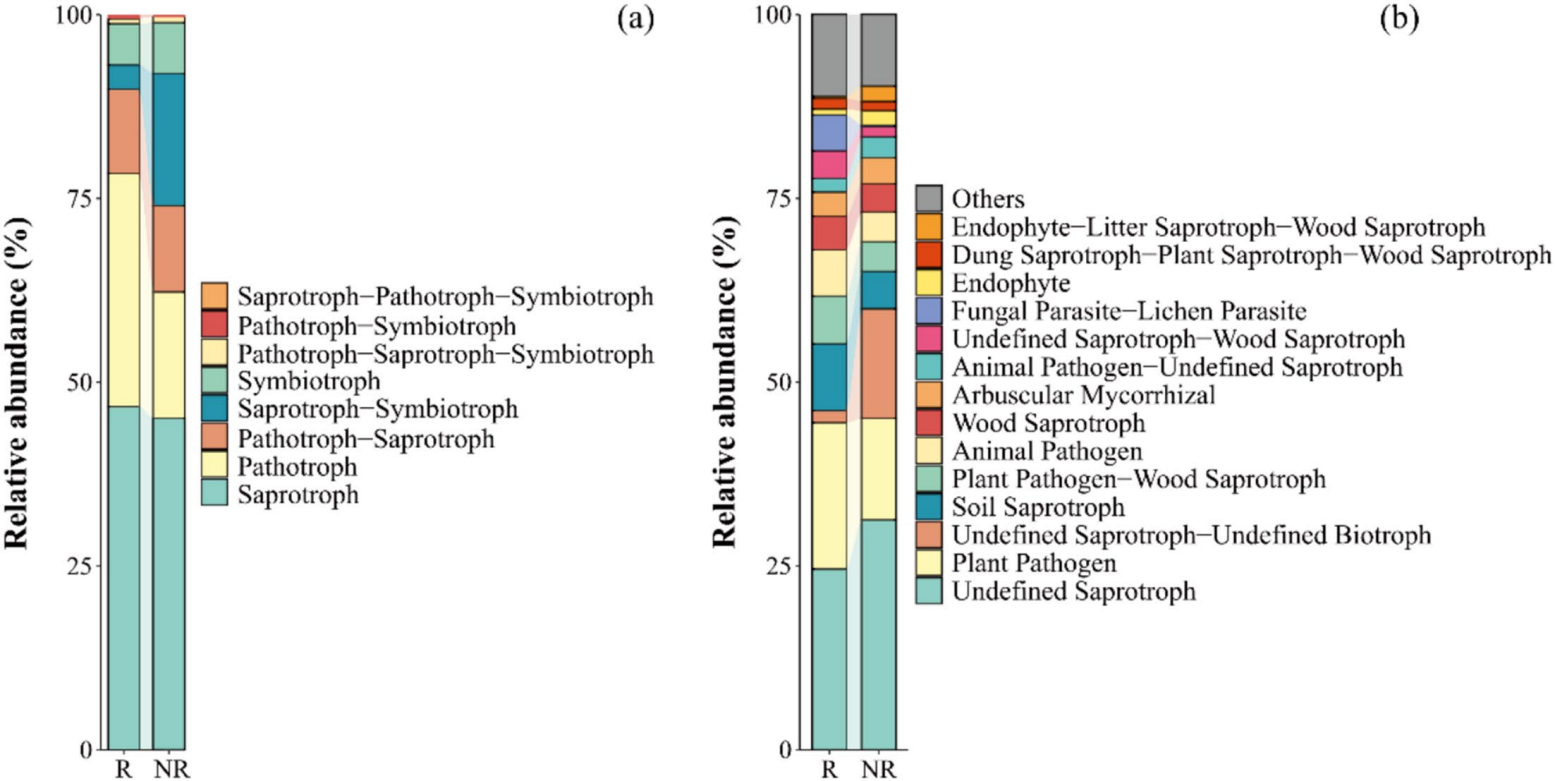
Relative abundance of different trophic modes **(a)** and guilds **(b)** in fungal communities. The trophic mode including 3 types: saprotroph, pathotroph, and symbiotroph. The guild has 12 types: animal pathogens, arbuscular mycorrhizal fungi, ectomycorrhizal fungi, ericoid mycorrhizal fungi, foliar endophytes, lichenicolous fungi, lichenized fungi, mycoparasites, plantpathogens, undefined root endophytes, undefined saprotrophs and wood saprotrophs. R: rhizosphere fungal community, NR: non-rhizosphere fungal community.

Based on the trophic mode (Figure 2a), the relative abundance of saprotrophs, pathotrophs, and pathotroph–saprotrophs were high in both the rhizosphere and non-rhizosphere fungal communities. For guilds (Figure 2b), both undefined saprotrophs and plant pathogens had a higher relative abundance, and the relative abundance of undefined saprotrophs in the rhizosphere fungal community was significantly lower than that in the non-rhizosphere fungal community (*p* < 0.05).

### 3.2 Diversity analysis of fungal communities

**Figure 3:**
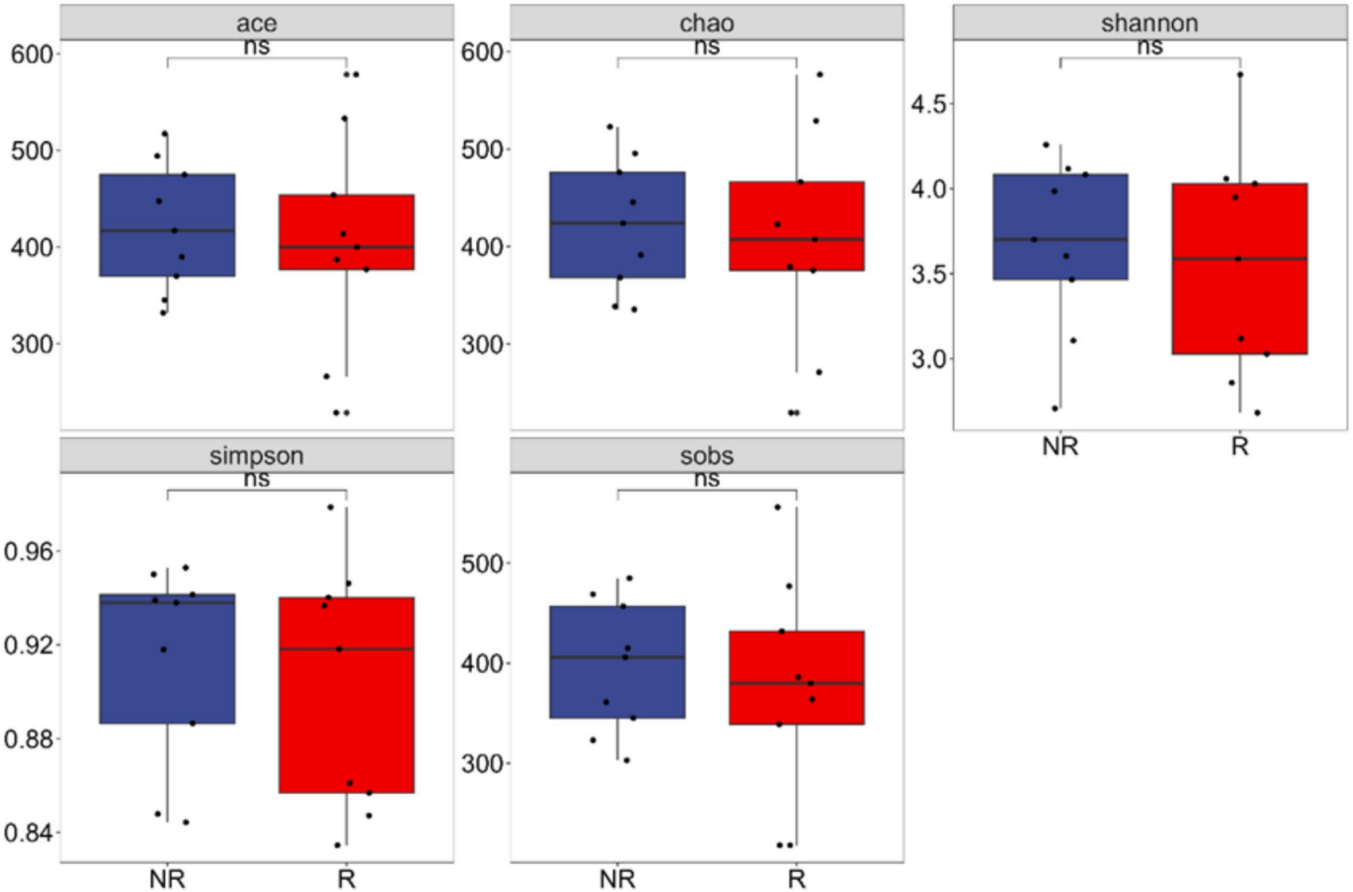
Fungal alpha diversity between the rhizosphere and non-rhizosphere of *Alsophila spinulosa*. Ace: abundance-based coverage estimator; sobs: species observed; R: rhizosphere fungal community, NR: non-rhizosphere fungal community; ns indicates that the difference is not significant (*p* > 0.05).

The richness and diversity of microbial communities were reflected by α-diversity analysis. The Ace, Chao, Shannon, Simpson, and Sob of fungal communities in the rhizosphere and non-rhizosphere soils of *A. spinulosa* did not show significant differences (*p* > 0.05). Therefore, the number and distribution of species were similar between the two communities (Figure 3).

**Figure 4:**
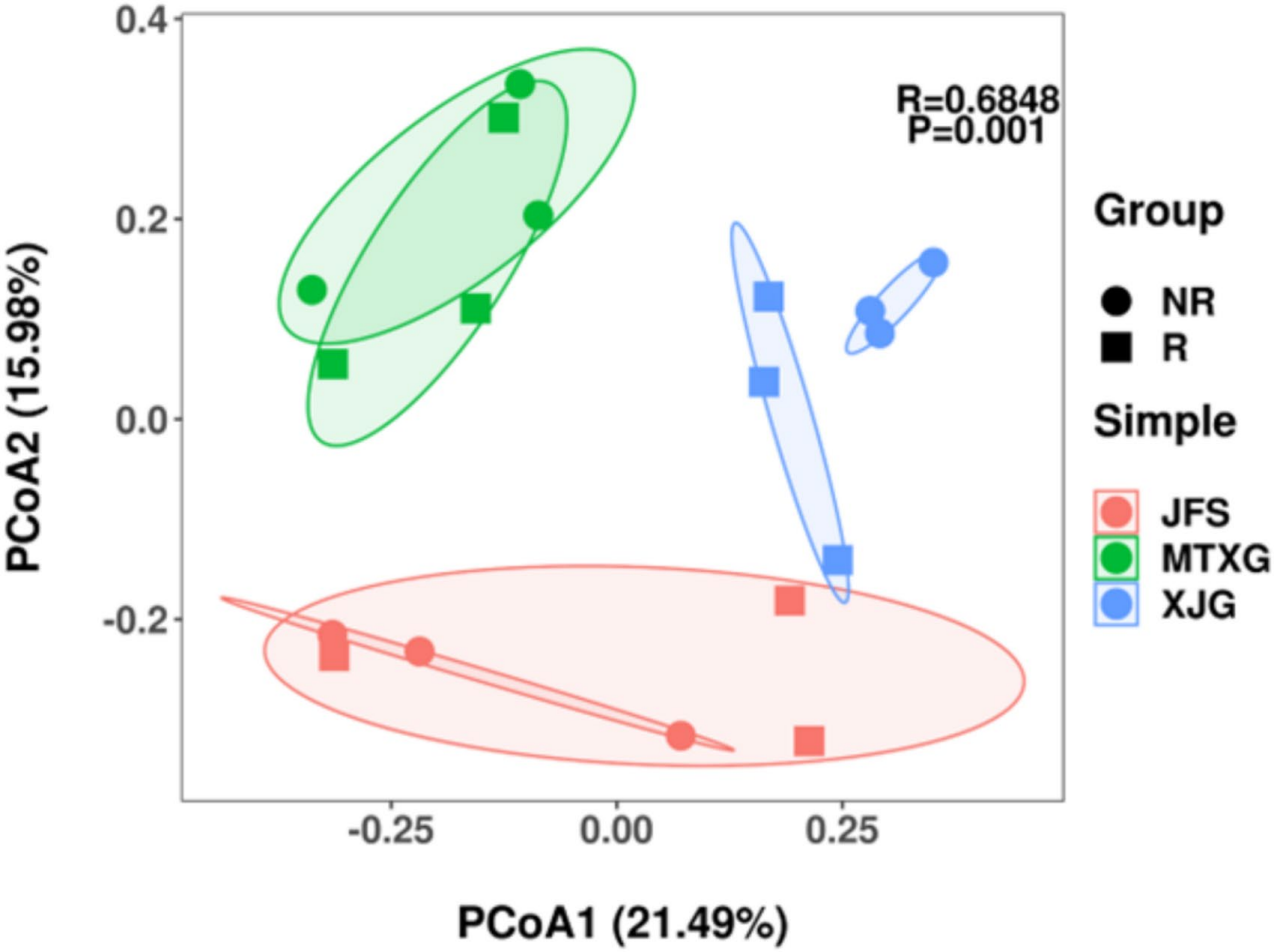
PCoA of fungal community composition based on the Bray–Curtis distance. R: rhizosphere fungal community, NR: non-rhizosphere fungal community; JFS: Jinfeng Mountain; MTXG: Mentouxi Gou; XJG: Xujia Gou.

Based on the Bray–Curtis distance, PCoA was used to determine the degree of difference in rhizosphere and non-rhizosphere fungal communities of *A. spinulosa* with different habitats (Figure 4). Fungal communities from different habitats were significantly separated, but only the rhizosphere and non-rhizosphere fungal communities in XJG were separated in the same habitat.

### 3.3 Difference analysis of fungal composition

**Figure 5:**
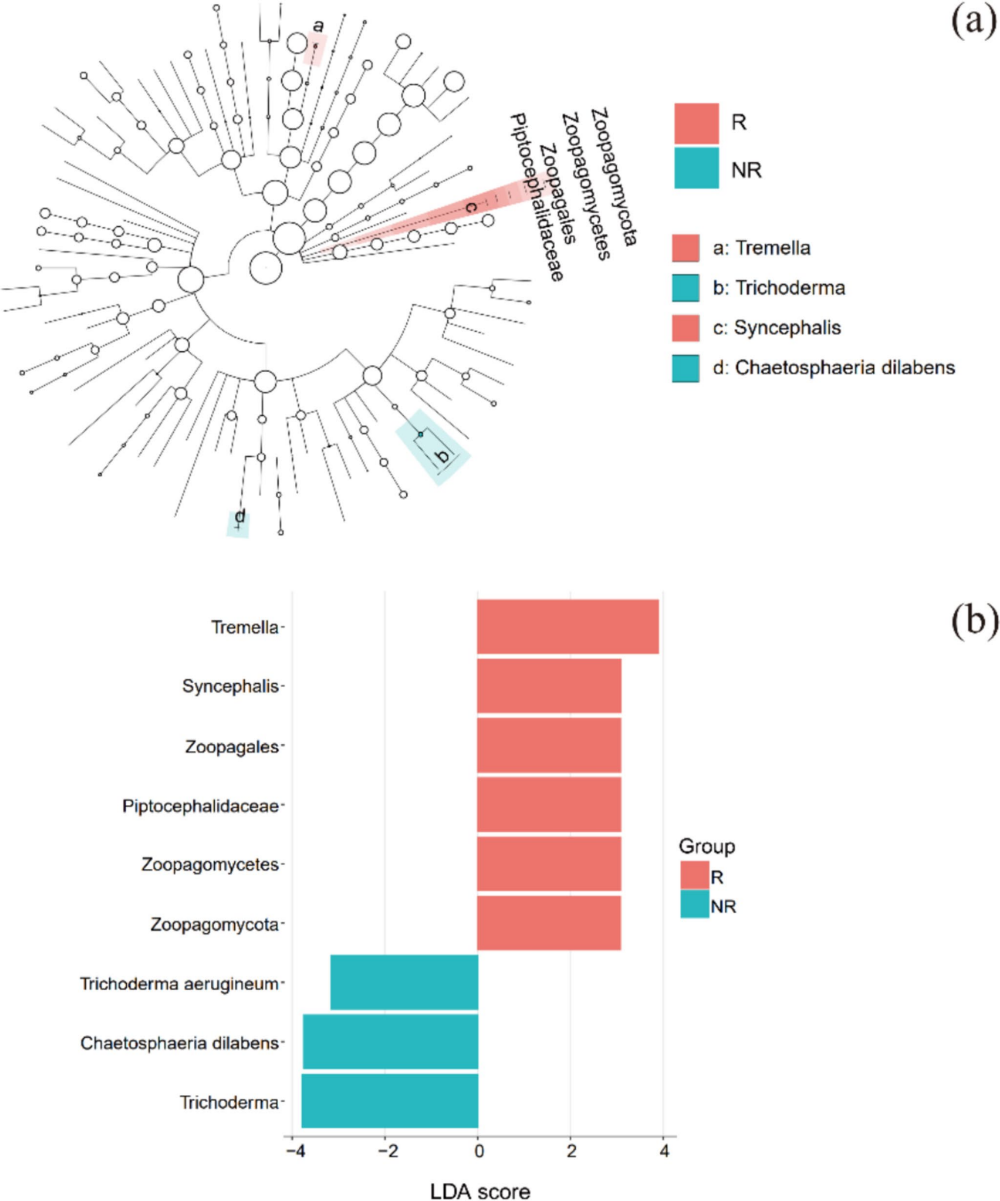
LEfSe analysis: **(a)** evolutionary branch diagram and **(b)** distribution histogram. R: rhizosphere fungal community, NR: non-rhizosphere fungal community.

LEfSe identified biomarkers with significant differential impacts. Biomarkers in the rhizosphere and non-rhizosphere fungal communities appeared at both ends of the evolutionary tree, but biomarkers from the same group were close to each other on the evolutionary tree (Figure 5a). This suggests that the biomarkers in the same group had closer genetic relationships and more similar functions and that the biomarkers in the two groups that were more distant had different functions.

At the phylum level, the biomarker in the rhizosphere was *Zoopagomycota*, a group of fungal parasites. At the genus level, biomarkers in the rhizosphere were *Arthopyrenia*, *Hypochnicium*, *Tremella*, and *Syncephalis*. Based on the FUNGuild results, *Arthopyrenia* was lichenized, meaning that it formed a symbiosis with algae and that they collectively formed a lichen. *Hypochnicium* was an undefined saprotroph, and *Tremella* was a fungal or lichen parasite. *Syncephalis* was a fungal parasite (Figure 5b).

There were only two biomarkers for non-rhizosphere fungal communities at the genus level: *Chaetosphaeria* and *Trichoderma*. *Chaetosphaeria* was an endophyte, litter saprotroph, or wood saprotroph, and *Trichoderma* was an undefined saprotroph.

**Figure 6:**
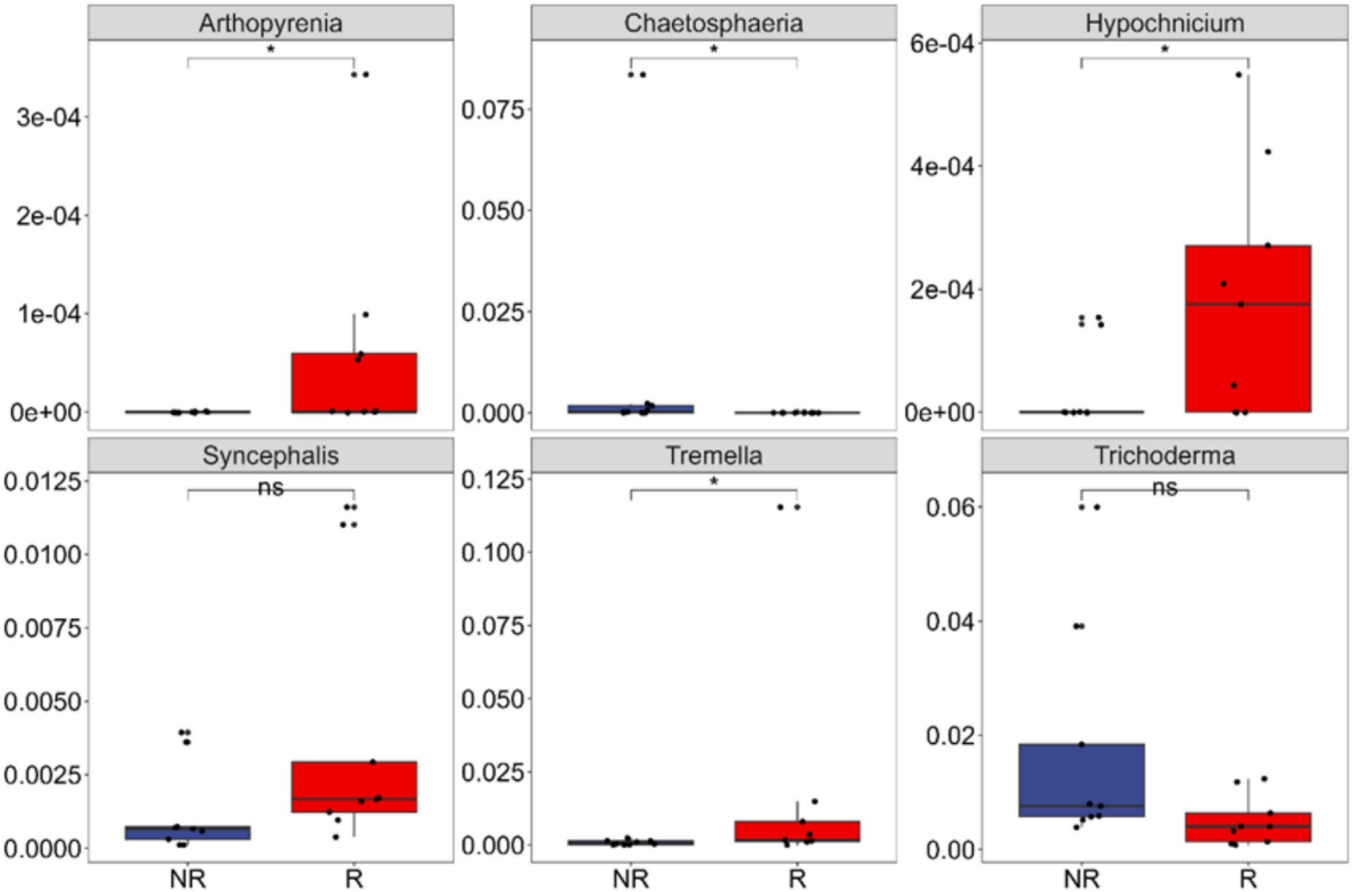
Differences in the relative abundance of each indicator species in rhizosphere (R) and non-rhizosphere (NR) soils at the genus level. *Arthopyrenia*, *Hypochnicium*, *Tremella*, and *Syncephalis* are biomarkers for rhizosphere fungal communities; *Chaetosphaeria* and *Trichoderma* are biomarkers for non-rhizosphere fungal communities. *0.01 < *p* ≤ 0.05; ns: *p* > 0.05.

The relative abundance of the above six biomarkers in rhizosphere (*Arthopyrenia*, *Hypochnicium*, *Tremella*, and *Syncephalis*) and non-rhizosphere (*Chaetosphaeria* and *Trichoderma*) fungal communities were compared. The relative abundance of these biomarkers was not high in the community. The highest in the rhizosphere community was *Tremella*, exceeding 1%, and the others were between 0.1 and 0.4%. The relative abundance of the 2 biomarkers in non-rhizosphere communities were both around 1% (Figure 6).

### 3.4 Core fungi and endemic species

**Figure 7:**
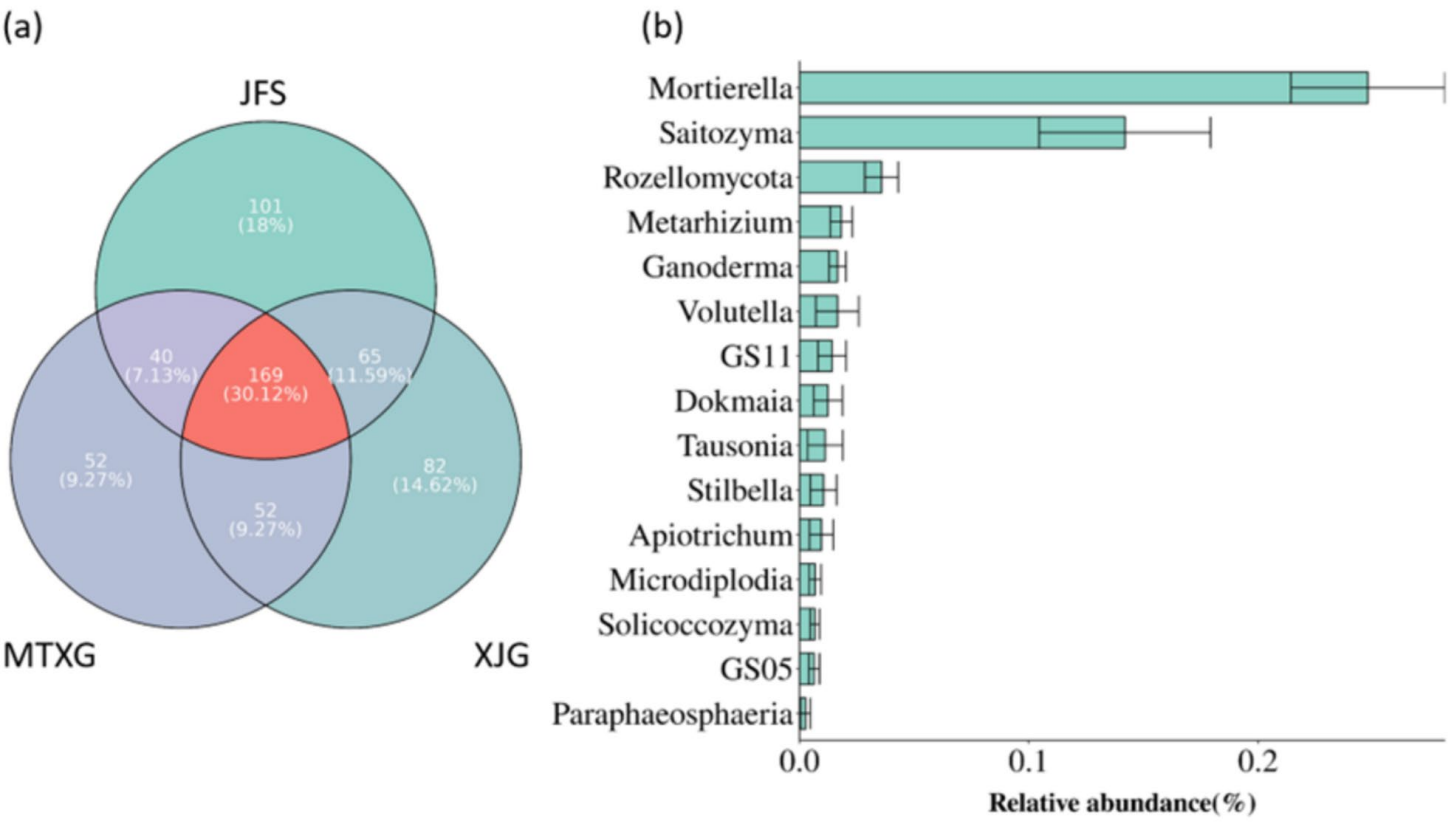
**(a)** Core fungi of rhizosphere fungal communities in three plots. **(b)** Top 15 most abundant genera among core fungi. JFS: Jinfeng Mountain; MTXG: Mentouxi Gou; XJG: Xujia Gou.

The core microbiome is considered a key component of the basic functions of holobionts (Dong et al., 2019). The genera that appeared in in all 3 habitats were the core fungi of the *A. spinulosa* rhizosphere soil fungal community; these 169 genera accounted for 30.12% of the genera in the rhizosphere community (Figure 7a). The most relatively abundant genera of the core fungi in the rhizosphere community were *Mortierella* and *Saitozyma*, which were in the wood saprotroph and fungal parasite/undefined saprotroph guilds, respectively (Figure 7b).

**Figure 8:**
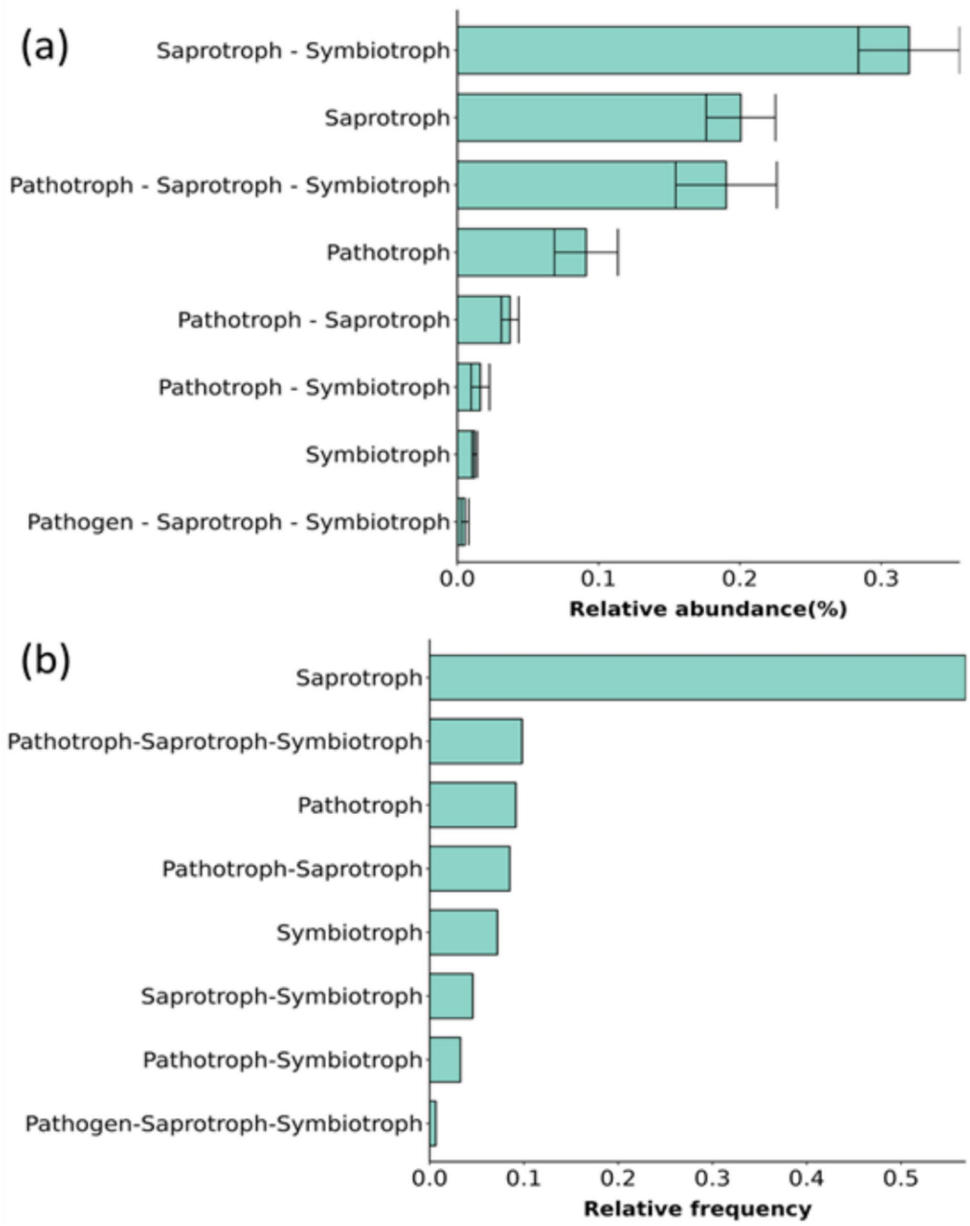
Relative abundance **(a)** and frequency **(b)** of different trophic types in rhizosphere core fungi.

The core fungi, comprising 169 genera, in the rhizosphere fungal community of *A. spinulosa* were divided into 8 functional types: saprotroph, symbiotroph, pathotroph, saprotroph/symbiotroph, pathotroph/symbiotroph, pathotroph/saprotroph, pathogen/saprotroph/symbiotroph, and pathotroph/saprotroph/symbiotroph. In terms of abundance, there were more fungi with saprotrophic and symbiotic nutritional characteristics, but in terms of frequency of occurrence, saprotrophic fungi were the most common (Figure 8).

**Figure 9:**
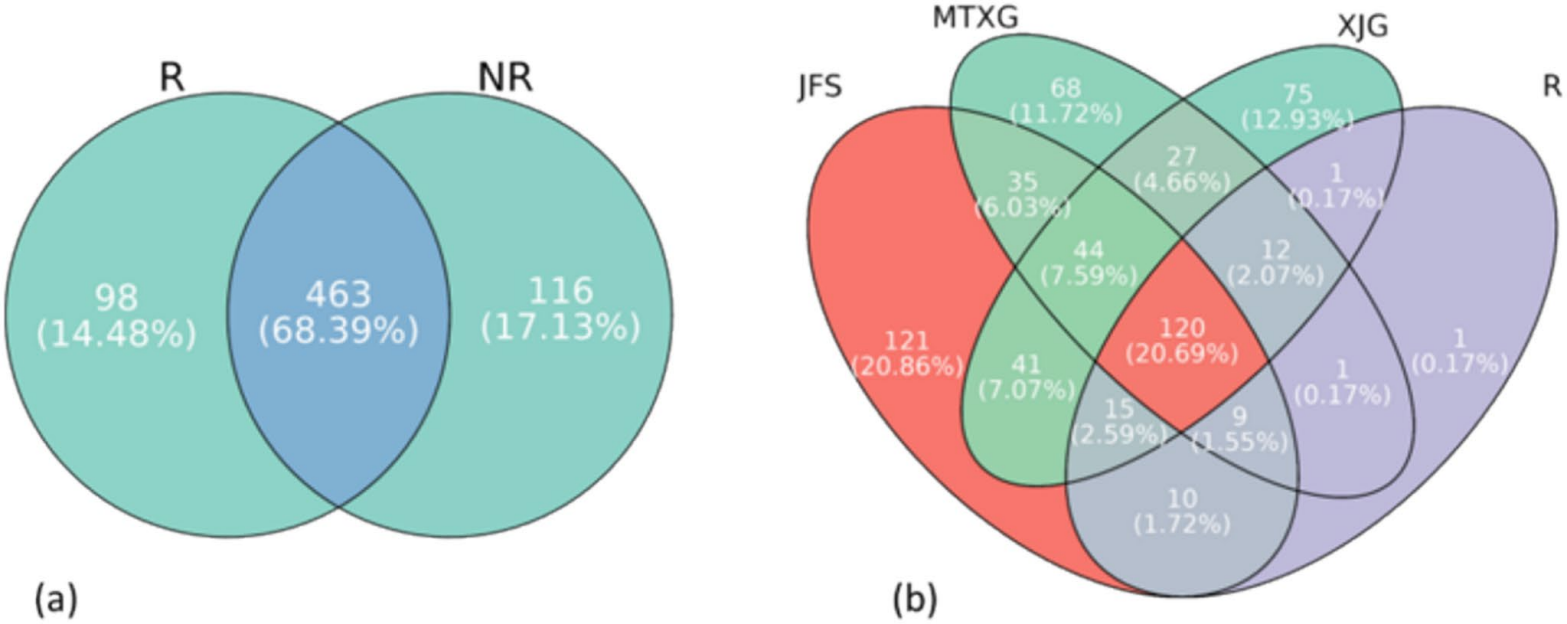
**(a)** Venn diagram of rhizosphere and non-rhizosphere soil fungi. R: rhizosphere fungal community, NR: non-rhizosphere fungal community. **(b)**. Venn diagram of three non-rhizosphere habitats and rhizosphere soil fungi. JFS: Jinfeng Mountain; MTXG: Mentouxi Gou; XJG: Xujia Gou; R: the fungal genus within the rhizosphere fungal community.

Comparing all fungi present in the *A. spinulosa* rhizosphere those not in the rhizosphere revealed 98 unique genera in the rhizosphere community, accounting for 14.48% of the total (Figure 9a). Among these unique genera, those with a higher relative abundance were *Mycenella*, *Myrothecium*, and *Henningsomyces*. *Mycenella* was in the symbiotroph guild, and *Myrothecium* and *Henningsomyces* were in the saprotroph guild.

The core fungi in the rhizosphere community were compared with those in non-rhizosphere communities. One genus, *Flavodon*, was found in the rhizosphere community but not in the non-rhizosphere community (Figure 9b), and its guild was undefined saprotroph.

### 3.5 Community assembly of rhizosphere and non-rhizosphere fungi

**Figure 10:**
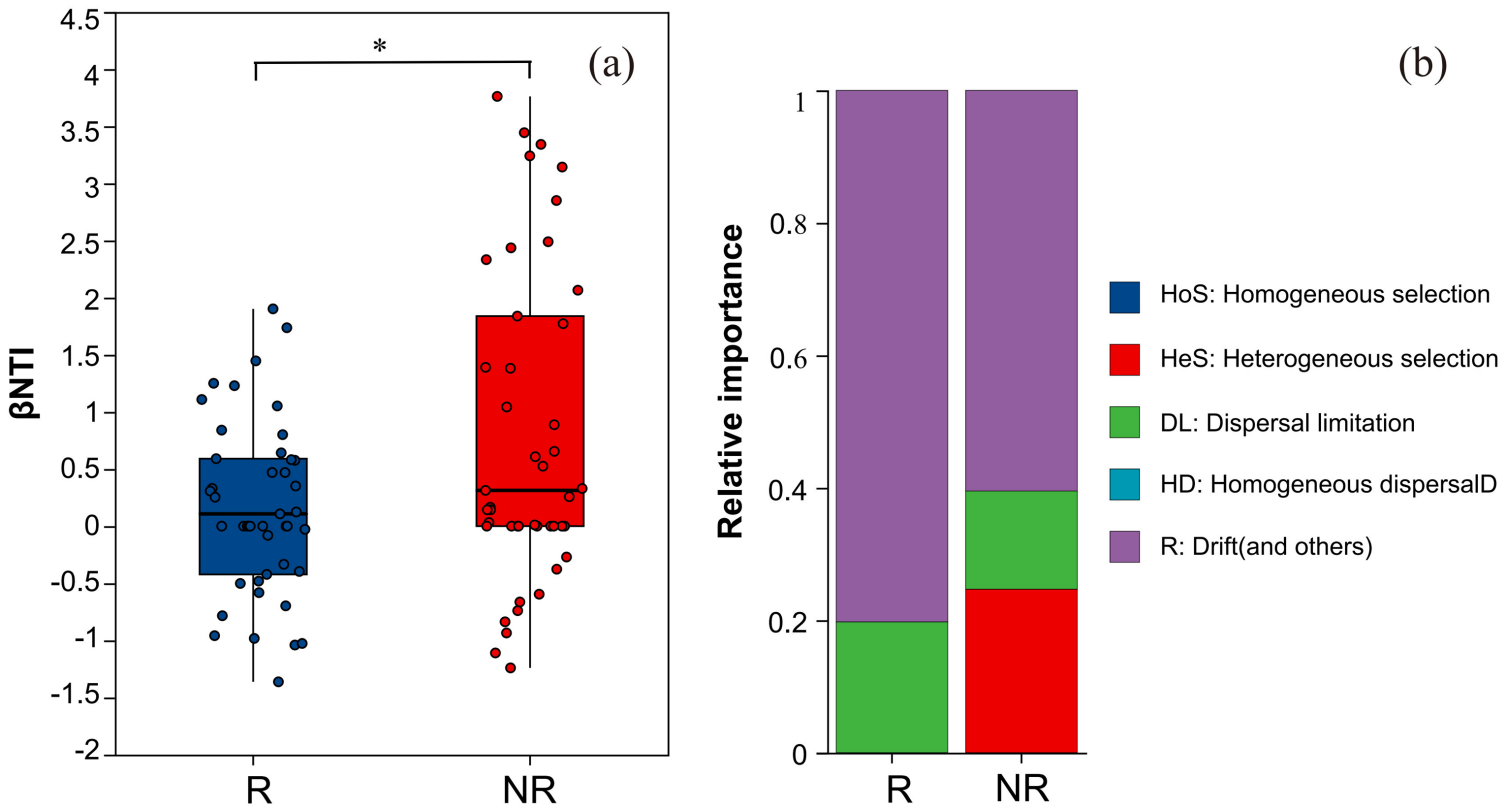
Analysis of community construction of rhizosphere and non-rhizosphere fungal communities in *Alsophila spinulosa*. **(a)** differences in βNTI between R and NR, **(b)** composition of ecological processes in R and NR. R: rhizosphere fungal community, NR: non-rhizosphere fungal community.

To evaluate the relative importance of certainty and randomness in the microbiome assembly, the β-closest taxon index (βNTI) and Raup–Crick index (RCBray) was calculated to infer the process of fungal community assembly changes in the rhizosphere and non-rhizosphere soil of *A. spinulosa*. The process of fungal community assembly in the rhizosphere and non-rhizosphere of *A. spinulosa* was significantly different (Figure 10a). Although both communities were dominated by random processes, there were more random components in the rhizosphere community (Figure 10b).

## 4 Discussion

### 4.1 Characteristics of rhizosphere and non-rhizosphere fungal communities

Ferns are the most primitive group of vascular plants. Although they have real roots, they are usually adventitious roots, and the root branch structure is simple with an underdeveloped vascular tissue (McAdam and Brodribb, 2012; Xiang et al., 2022). Mycorrhizal fungi often form symbiotic relationships with plants and have complex interactions with host plants and the environment, and this relationship is critical for plant growth and soil ecosystem health (Qu et al., 2023; Reese et al., 2018; Shao et al., 2021). In general, the richness and diversity of fungal communities in plant rhizosphere soil are higher than those in non-rhizosphere soil (Liu et al., 2021; Lu and Ang Zhang, 2006); however, this difference was not significant in the rhizosphere soil fungal community of *A. spinulosa*. After excluding sampling artifacts, these observed patterns likely reflect intrinsic biological characteristics of both the habitat and *A. spinulosa* itself. Both environmental stress and habitat homogenization may attenuate rhizosphere effects in plants—a hypothesis requiring further empirical validation through controlled studies. Furthermore, as an ancient relict species, *A. spinulosa* likely exhibits generalized symbiotic strategies with fungi rather than highly specific dependencies—a pattern consistent with early-diverging plant lineages’ microbial partnership characteristics.

### 4.2 Biomarkers in the rhizosphere community

In this study, four genera served as biomarkers for rhizosphere fungal communities. *Hypochnicium* is a genus of corticioid, wood-inhabiting fungi in Polyporales with a worldwide distribution. By decomposing wood, wood-inhabiting fungi release important elements, such as carbon, nitrogen, and phosphorus, into the soil and participate in nutrient cycling, promoting plant growth. *Tremella* and its accompanying fungi often decompose wood and promote nitrogen fixation. *Syncephalis* is a parasitic fungus that preys on protozoans, such as amoebas and nematodes, and inhabits them both internally and externally (Zhou, 2005). In summary, these fungi may play a role in providing nutrients and defending against diseases, but more research is needed to prove this. Both environmental stress and habitat homogenization may attenuate rhizosphere effects in plants—a hypothesis requiring further empirical validation through controlled studies.

### 4.3 Core and endemic fungi in the rhizosphere

Core microorganisms are closely related to the host and may have direct or indirect antagonism with other microorganisms, thus having positive or negative effects on plant growth (Busby et al., 2016; Matthew and Agler, 2016). The core fungi in the rhizosphere soil of *A. spinulosa* were dominated by saprophytes, and the most abundant genus, *Mortierella*, can be used to produce polyunsaturated fatty acids (Jin, 2016). At the same time, it also has an inhibitory effect on some plant pathogenic fungi (Yu et al., 2016). The second most abundant genus, *Saitozyma*, is a genus of the order *Tremella*les and is commonly found in soil and the plant rhizosphere (Leguina et al., 2019; Zhou et al., 2021; Zhou, 2020). These two genera can be regarded as beneficial fungi to a certain extent, but whether they have beneficial effects on *A. spinulosa* growth requires further research and verification.

Fungi endemic to the *A. spinulosa* rhizosphere (mentioned in Section 3.5) were mainly saprophytic fungi, and the most abundant were *Mycenella*, *Myrothecium*, and *Henningsomyces*. *Myrothecium* is widely found in plants and soils. Most species have strong cellulolytic abilities. Some species produce antibiotics, and some cause plant diseases (Wu, 1991). *Henningsomyces* is a wood-dwelling fungus that was first reported in China in 2007, and its specific functions have yet to be determined (Wei et al., 2007). Many dominant and unique saprophytic fungi in the *A. spinulosa* rhizosphere promote its growth to some extent, making them potential beneficial fungi.

The genus *Flavodon* was unique to the rhizosphere fungal community. Research has recently been conducted on *Flavodon*. *Flavodon* flavus is used in various industries for the degradation of lignin-related pollutants. *Flavodon* ambrosius can coexist with insects and grow on lignocellulose in dead trees. The cellulase activity of *Flavodon* sp. x10 is 1.388 U/mL, which is higher than that of the white rot fungi, *Ganoderma applanatum*, *Trametes hirsuta*, and *Fomes fomentarius*, giving it a significant advantage in the development and utilization of lignocellulose (Wang et al., 2023).

### 4.4 Community assembly of rhizosphere fungi

Based on βNTI and RCBray, the community construction process was divided into two deterministic processes, namely homogeneous selection and heterogeneous selection, and three stochastic processes, namely dispersal limitation, homogeneous dispersal, and drift and other ecological processes. The construction of the *A. spinulosa* rhizosphere fungal community was random and mainly affected by drift and other ecological processes. This shows that fungi are greatly affected by accidental events such as environmental changes, weather events, and habitat changes. One of the reasons why *A. spinulosa* is endangered is that it has strict habitat requirements. Environmental changes affect fungal communities, which may adversely impact the growth of *A. spinulosa* to some extent. In addition, the current insufficient or unstable environmental resources will decrease the fungal population size, thus increasing its sensitivity to drift and making some fungi dominate or disappear, which changes the functions of the community.

## 5 Conclusion

The disparities between rhizospheric and non-rhizospheric fungal communities in *A. spinulosa* soils primarily manifested in species composition rather than taxonomic abundance. The two most abundant genera in the rhizospheric fungal community, *Mortierella* and *Saitozyma*, play pivotal roles in soil material cycling and ecological system formation. The enriched biomarker genera and rhizosphere-unique genera within the fungal community may play distinct functional roles in *A. spinulosa*’s adaptation to specific habitats, such as lignin degradation and symbiotic interactions. Stochastic dispersal processes—such as spore propagation and ecological drift—dominated the assembly of rhizospheric communities, whereas deterministic processes (e.g., environmental filtering) exerted stronger influences on non-rhizospheric communities. This study delineates the assembly principles and ecological signatures of *A. spinulosa*-associated fungal communities, providing both a theoretical foundation for utilizing unique fungal functions and empirical support for implementing plant–soil-microbe synergistic restoration strategies in *A. spinulosa* conservation efforts.

## Data Availability

The datasets presented in this study can be found in online repositories. The names of the repository/repositories and accession number(s) can be found at: https://www.ncbi.nlm.nih.gov/, PRJNA1033401.
